# In This Issue

**DOI:** 10.1111/cas.70147

**Published:** 2025-08-02

**Authors:** Kohsuke Takeda

**Affiliations:** ^1^ Nagasaki University Nagasaki Japan

Volume 116, Issue 8, August 2025

## Diversity of U1 Small Nuclear RNAs and Diagnostic Methods for Their Mutations

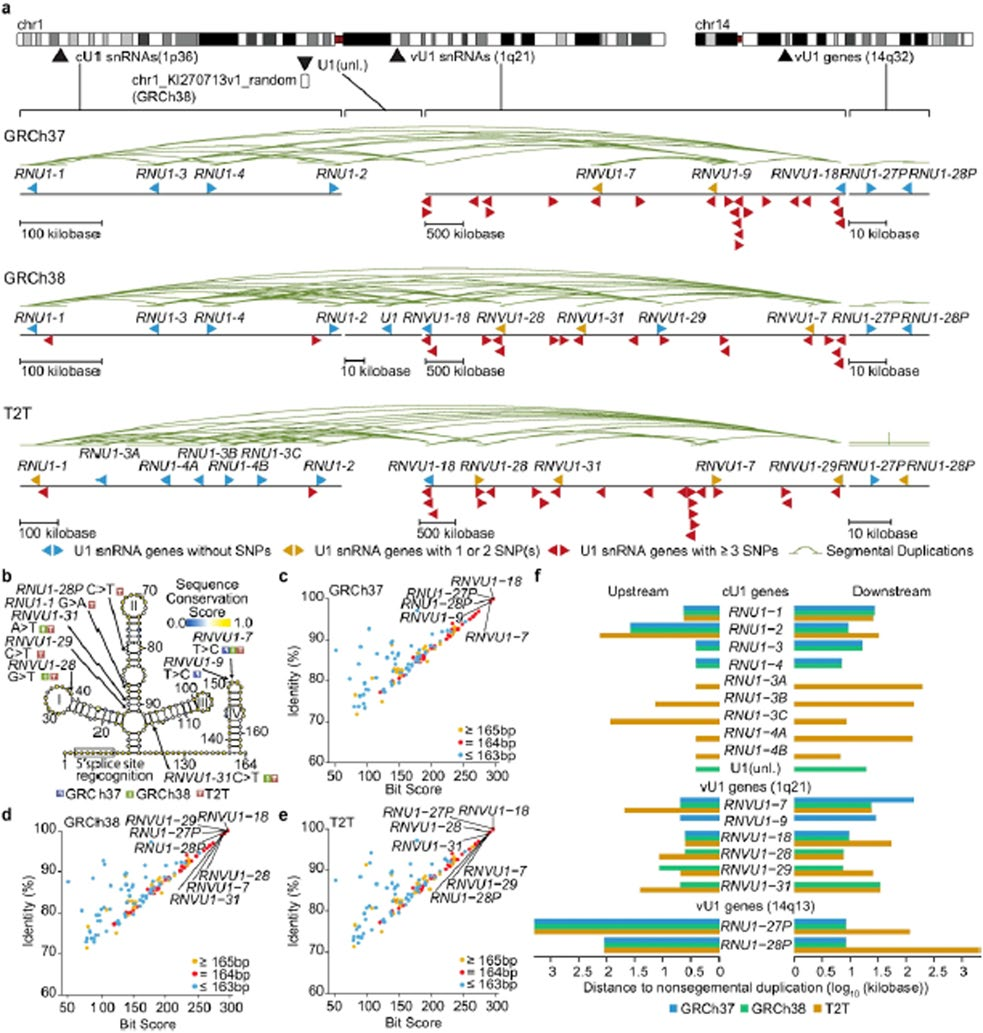

Some of the smallest pieces of our genetic code might play a big role in cancer, but for decades, many of these were a mystery. This scenario changed with the first gapless human genome, which filled in previously missing sections. Many of these regions contain stretches of DNA that are repeated and difficult to study. But these regions are now known to play important roles in various diseases. Within these regions lies a tiny but crucial molecule called U1 small nuclear RNA (snRNA). It doesn't make proteins, but it is essential for a process called splicing, which helps to remove unwanted segments of the RNA and use the genetic instructions carefully.

When U1 snRNA is mutated or undergoes a sudden change, this process can go off track, as it can cause cells to misread hundreds of genes, including the ones linked to cancer. These mutations have been found in brain tumors, blood, and liver cancers and may even activate pathways that worsen tumor growth. But finding them has been incredibly difficult, as they are buried deep within the repetitive DNA, where standard genetic tools often fail.

In this study, Nakashima et al. used a pangenome (a reference built from many people's genomes) and haploid genome (a reference built from a single set of chromosomes) analysis to uncover some of these parts of the U1 snRNA regions that had been missed. By using the pangenome in a graphical representation, the researchers were able to identify the genetic changes successfully. They discovered different types of genetic changes, including copy number (extra or missing copies of genes) and single‐nucleotide variations, even in places where they were not expected to matter.

They also applied new tools to detect these hidden mutations, which could help diagnose cancers earlier and more accurately. This is important because these mutations are often linked to poor outcomes in cancers such as brain tumors and blood cancer, so finding them early could help guide treatment.

https://onlinelibrary.wiley.com/doi/full/10.1111/cas.70110

## OCT‐2 Is Associated With Pro‐Metastatic Epigenomic Properties of Triple‐Negative Breast Cancer Cells

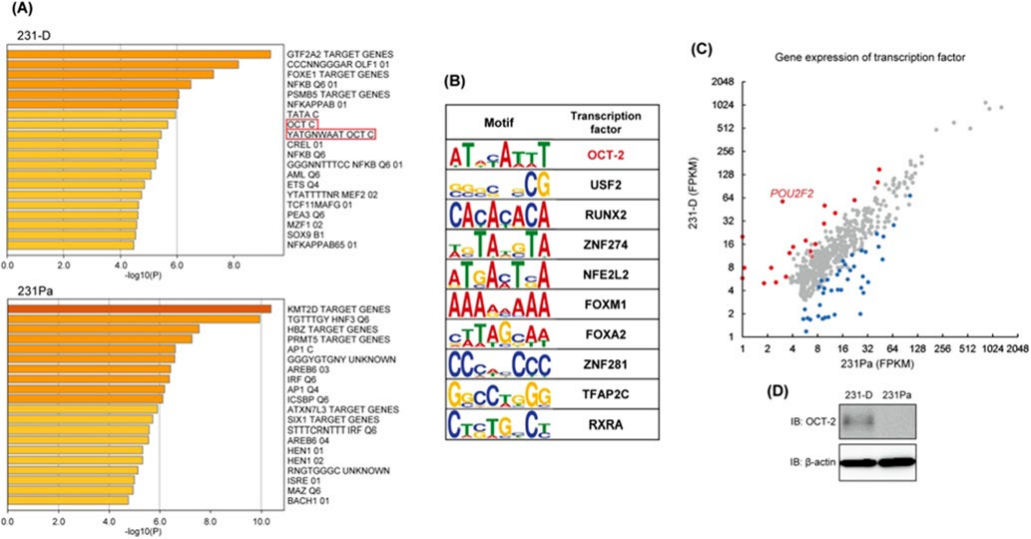

Triple‐negative breast cancer (TNBC) is an aggressive form of breast cancer. Due to the lack of three common receptors—estrogen receptor, progesterone receptor, and HER2—which are often targeted in standard breast cancer treatments, TNBC is mainly treated with traditional chemotherapy. Unfortunately, TNBC tends to be resistant to these drugs, creating an urgent need for new treatment strategies.

Massively parallel DNA sequencing technology has helped scientists identify specific regions of DNA that regulate gene activity. Transcription factors can bind to these regions to either activate gene expression or repress it, and this process accompanies epigenetic changes. Researchers are exploring ways to target these regulatory processes to develop new treatment strategies for cancer. One key discovery is the presence of super‐enhancers—clusters of enhancers, and these super‐enhancers can dramatically increase the expression of genes that drive cancer growth and metastasis, often serving as binding hubs for transcription factors. However, the role of super‐enhancers and epigenetic regulation in TNBC progression is not fully understood.

To address this gap, Ogikubo et al. used a TNBC cell line (MDA‐MB‐231 cells) and its metastatic variant cell line (MDA‐MB‐231‐5a‐D cells). Their analysis revealed the presence of specific super‐enhancers in MDA‐MB‐231 cells and MDA‐MB‐231‐5a‐D cells, as well as some common super‐enhancers in both cell lines. These super‐enhancers were located close to genes that are involved in conferring malignant characteristics to cancer cells. The transcription factor OCT‐2 was highly expressed in metastatic cells and was associated with super‐enhancer formation. OCT‐2 increased the expression of malignancy‐related genes by inducing epigenetic changes. Also, forced expression of OCT‐2 increased the metastasis of TNBC cells in mice. Supporting this, OCT‐2 was also found to be highly expressed in human TNBC cases. These findings suggest that OCT‐2 may not only help predict how TNBC progresses but could also be a promising target for future treatments.

https://onlinelibrary.wiley.com/doi/full/10.1111/cas.70093

## Clonal Hematopoiesis and Solid Cancers

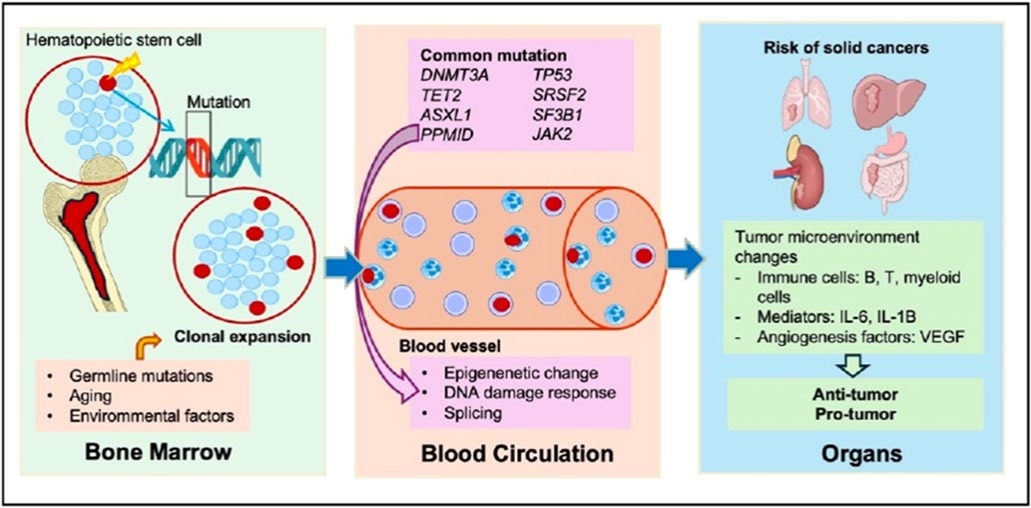

As people age, certain blood‐forming cells in the bone marrow can acquire changes in their genetic material. These changes may give one cell an advantage, allowing it to multiply more than others. This process is known as clonal hematopoiesis, and when it occurs without signs of disease, it is referred to as clonal hematopoiesis of indeterminate potential (CHIP). Although often harmless, CHIP has been linked to an increased risk of blood cancers—and now, growing evidence suggests it may also influence the development of solid tumors.

In this issue, Nguyen et al. review the emerging link between CHIP and solid tumors such as those of the lung, breast, stomach, and colon. They note that CHIP and solid cancers share many risk factors, including aging, inherited genetic traits, environmental exposures, long‐term inflammation, and lifestyle factors such as smoking and poor diet. Importantly, mutations commonly found in CHIP—including *DNMT3A*, *TET2*, *ASXL1*, and *TP53*—have also been detected in a range of solid tumors. These appear at varying frequencies depending on the cancer type, with stronger associations observed in lymphomas, lung cancer, and breast cancer. The authors also report that mutations affecting DNA structure are more frequent in stomach and bladder cancers, while gene processing mutations are more common in colon and head and neck cancers.

Experimental studies suggest that CHIP may affect cancer progression by altering the tumor environment. It may reduce the immune system's ability to fight tumors, promote inflammation, and support the growth of new blood vessels that feed the tumor. These changes can make it easier for tumors to grow and spread. Clinically, CHIP has been linked to poorer responses to cancer treatment, higher mortality, and an increased risk of therapy‐related complications such as heart disease and secondary cancers.

These findings suggest that CHIP may play a more active role in cancer development than previously thought. Understanding how CHIP contributes to cancer biology, treatment response, and long‐term outcomes could help improve cancer risk prediction and inform more tailored treatment strategies in the future.

https://onlinelibrary.wiley.com/doi/full/10.1111/cas.70097

